# Training of clinical reasoning with a Serious Game versus small-group problem-based learning: A prospective study

**DOI:** 10.1371/journal.pone.0203851

**Published:** 2018-09-11

**Authors:** Angélina Middeke, Sven Anders, Madita Schuelper, Tobias Raupach, Nikolai Schuelper

**Affiliations:** 1 Division of Medical Education Research and Curriculum Development, Study Deanery of University Medical Centre Göttingen, Göttingen, Germany; 2 Department of Legal Medicine, University Medical Centre Hamburg-Eppendorf, Hamburg, Germany; 3 Department of Haematology and Medical Oncology, University Medical Centre Göttingen, Göttingen, Germany; 4 Department of Cardiology and Pneumology, University Medical Centre Göttingen, Göttingen, Germany; 5 Health Behaviour Research Centre, University College London, London, United Kingdom; Waseda University, JAPAN

## Abstract

**Introduction:**

Serious Games are increasingly being used in undergraduate medical education. They are usually intended to enhance learning with a focus on knowledge acquisition and skills development. According to the current literature, few studies have assessed their effectiveness regarding clinical reasoning (CR). The aim of this prospective study was to compare a Serious Game, the virtual Accident & Emergency department ‘EMERGE’ to small-group problem-based learning (PBL) regarding student learning outcome on clinical reasoning in the short term.

**Methods:**

A total of 112 final-year medical students self-selected to participate in ten 90-minute sessions of either small-group PBL or playing EMERGE. CR was assessed in a formative examination consisting of six key feature cases and a final 45-minute EMERGE session.

**Results:**

Overall, the EMERGE group (n = 78) scored significantly higher than the PBL group (n = 34) in the key feature examination (62.5 (IQR: 17.7)% vs. 54.2 (IQR: 21.9)%; p = 0.015). There was no significant difference in performance levels between groups regarding those cases which had been discussed in both instructional formats during the training phase. In the final EMERGE session, the EMERGE group achieved significantly better results than the PBL group in all four cases regarding the total score as well as in three of four cases regarding the final diagnosis and the correct therapeutic interventions.

**Conclusion:**

EMERGE can be used effectively for CR training in undergraduate medical education. The difference in key feature exam scores was driven by additional exposure to more cases in EMERGE compared to PBL despite identical learning time in both instructional formats. EMERGE is a potential alternative to intensive small-group teaching. Further work is needed to establish how Serious Games enhance CR most effectively.

## Introduction

Functioning independently in clinical practice requires competencies related to clinical reasoning (CR), including making informed choices about diagnostic procedures, identifying the correct diagnosis and recommending the most appropriate therapy [1]. At least two processes form the basis of CR: the intuitive and the analytical approach [2]. The intuitive approach proves effective most of the time (e.g. by using pattern recognition and inductive logic), but solving more complex cases, e.g. when a patient´s symptoms are not readily recognized as belonging to a specific illness category, requires analytical processing (e.g. hypothetico-deductive reasoning; deliberate and purposeful thinking) [2]. However, novices frequently apply analytical problem-solving processes as they do not yet recognize clinical patterns. With increasing experience and expertise in problem-solving, novices acquire the ability of quick pattern recognition and intuitive CR.

It is one of the most important but challenging core tasks of undergraduate medical education to enable students to make a correct diagnosis and to initiate adequate therapeutic measures [1,3].

Approaches to curriculum development put great emphasis on the alignment between learning objectives, educational strategies and assessment methods [4]. Traditional lectures may be suitable for addressing learning objectives at the lowest level (factual knowledge). In contrast, conveying complex learning objectives requires different educational strategies such as small-group or bedside teaching. Such methods may encourage CR, yet they are very resource intensive regarding staff time [5] and, consequently, costs [6]. Amongst others, their effectiveness depends on the group size and the competence of the tutor [7]. Furthermore, standardising the content of small-group teaching sessions may be difficult. The learning objectives covered in bedside teaching, for instance, depend on the patients who are treated on the wards [8]. Consequently, students may be less likely to encounter patients with contagious or rare diseases and life-threatening symptoms as these can hardly be involved in clinical teaching. Moreover, there is an obligation to provide optimal patient treatment and to ensure maximum safety which creates a fundamental ethical tension if real patients are asked to support the training of medical students in emergency situations [9].

The rapid growth of digital infrastructure and the increasing availability of computer and internet services at medical schools facilitate the development of innovative formats fostering CR in medical education. Amongst the wealth of digital teaching interventions available to medical educators, Serious Games (i.e., computer games designed for a serious purpose rather than pure entertainment) are already being used at a number of medical schools worldwide [10]. Most of these games are intended to enhance learning with a focus on knowledge acquisition and skills development [11,12]. For example, students might be given the possibility to encounter virtual patients and practice skills that are required for the adequate treatment of patients (e.g. conducting an anamnesis, diagnostic measures and therapeutic procedures; decision making; triaging). Presenting students with a variety of virtual patients might engage them in deliberate practice [13], an important factor for the training of CR [1] and the acquisition of expert performance [14]. Moreover, by means of virtual patients, learning demands as well as context and content complexity can be aligned to the students´ individual needs and capabilities and students are enabled to work at their own pace [13,15].

Previous work indicates that students are very motivated to use Serious Games because they are perceived as more engaging and interactive than traditional learning methods and as being a useful aid for knowledge consolidation [16–19]. Students especially appreciate that Serious Games offer them a high degree of autonomy and independence in their learning and the possibility to work through a complete patient problem on their own [17]. Serious Games enable students to improve their diagnostic problem-solving skills [17], a cognitive function crucially needed for successful CR. Students learn to treat various patients simultaneously and, unlike in real clinical scenarios, errors can be allowed or even be made on purpose. This way, students can explore their implications and learn how to manage the consequences while not causing any harm to real patients [9,20]. Serious Games thus offer the possibility to teach and learn in a patient-centred way where live patients are not available or must not be put at risk. Moreover, educational content and delivery can be highly standardized in order to ensure that all students are exposed to the same content [21]. Although Serious Games seem to bring a benefit to medical education, there is only scarce statistical evidence supporting this assumption. Little research has been done and publications focussing on the effectiveness in comparison to traditional teaching formats rather than simply reporting student satisfaction with Serious Games are rare.

Therefore, the primary aim of this study was to compare a Serious Game, the virtual A&E department ‘EMERGE’ to small-group problem-based learning (PBL) regarding student learning outcome on clinical reasoning in the short term.

We hypothesised that student learning outcome would be higher in the EMERGE group given that students work more independently in this format.

## Materials & methods

### Study design

In summer term 2016, this monocentric, prospective study was carried out at Göttingen Medical School. Its six-year undergraduate curriculum consists of two preclinical years, three clinical years and a practice year. This study included fifth-year medical students who were enrolled in a six-week repetition module and consisted of a six-week intervention phase followed by a formative assessment.

All fifth-year medical students were required to take ten 90-minute teaching sessions of CR. They self-selected to participate in either small-group PBL or playing EMERGE during these ten sessions. Participation in one of the two teaching formats was mandatory for all students. PBL groups consisted of six to eight students each who discussed five different cases in detail supported by peer instructors, thus fostering deep learning. Two sessions were spent working on each case and the cases were discussed independently from one another. Students were asked to spend some leisure time between the two classroom sessions on self-directed learning about the teaching content covered in the cases. During classroom sessions, a 15-minute plenary discussion was organised for each of the five cases with a clinician available to answer and clarify questions. In contrast, students in the EMERGE group (approximately 25 students in one computer room) worked on up to ten different cases per session at their own pace with an experienced clinician available to clarify any questions. In total, the EMERGE group was exposed to more than 40 different cases belonging to the following content areas of internal medicine: cardiology/pulmology, nephrology/rheumatology, gastroenterology/endocrinology and haematology/oncology. Students were exposed to each case up to five times during the training phase. The cases used in EMERGE sessions were written by two of the authors (MS & NS) and are not included in the commercially available version of the game.

While learning time was kept similar in both formats and set by the curricular requirements (ten 90-minute sessions of CR teaching), the number of different clinical cases each student was exposed to was eight times higher in the EMERGE group (40 cases) than in the PBL group (5 cases). The concept of PBL provided for a detailed learning approach with two classroom sessions being spent on each case, thus limiting the total number of cases that were presented during the training phase to five. Students in the EMERGE group, in contrast, were exposed to more content in the same time which was one of the rationales underlying the above hypothesis that learning outcome would be higher in the EMERGE group than in the PBL group.

After six weeks of intervention, CR was assessed in a formative key feature examination comprising six cases and a gaming session during which four patient cases were presented.

In the first part of the exit examination, the students had to answer 24 key feature questions on diagnostic procedures and therapeutic options, relating to six different cases. All questions had to be answered using the long-menu format. In this format, students have to generate a spontaneous answer of which at least three letters need to be entered into an answer field. They are then able to view a long alphabetical list of response options containing the corresponding three letters and to choose the intended answer from the list. The alphabetical lists had already been used and tested in other research projects conducted by this research group. For a detailed description of the type of key feature cases used in our studies please see Ludwig et al. [22]. One key feature question used in the current trial was the following:

“A 45-year old women (170 cm, 56 kg) reports to Accident & Emergency because of palpitations. She says that she has repeatedly been suffering from hot flashes and sweating and that she felt irritable at times. At first, she believed this was a sign of incipient menopause. For the past few hours, she has had palpitations, and this caused her to report to the hospital. The complete medical history and physical examination do not provide any hints to the underlying disease. The patient appears to be concerned but not agitated. Vital signs are normal except for a tachycardia (heart rate 110/min). Which diagnostic test should immediately be done in this patient?” (expected answer: electrocardiogram)

Two key feature cases (‘Hodgkin lymphoma’, ‘sarcoidosis’) referred to content that was discussed in both the EMERGE and the PBL groups; four other cases (‘heart failure’, ‘hyponatremia’, ‘hyperthyroidism’, ‘fever in aplasia’) were related to content that only students in the EMERGE group had been exposed to during the training phase.

In the second part of the examination, all students were presented with four unknown cases in EMERGE: ‘Non-ST-elevation myocardial infarction’ (‘NSTEMI’), ‘pancreatitis’, ‘gastrointestinal hemorrhage’ and ‘asthma exacerbation’. None of these cases had previously been discussed during the training phase in any group. While playing EMERGE, all gaming activities were saved in log files automatically. All EMERGE log files were examined in order to analyse procedural aspects such as how students approached taking a history, carrying out a physical exam, ordering lab tests and additional diagnostic tests.

### EMERGE

**Fig 1:**
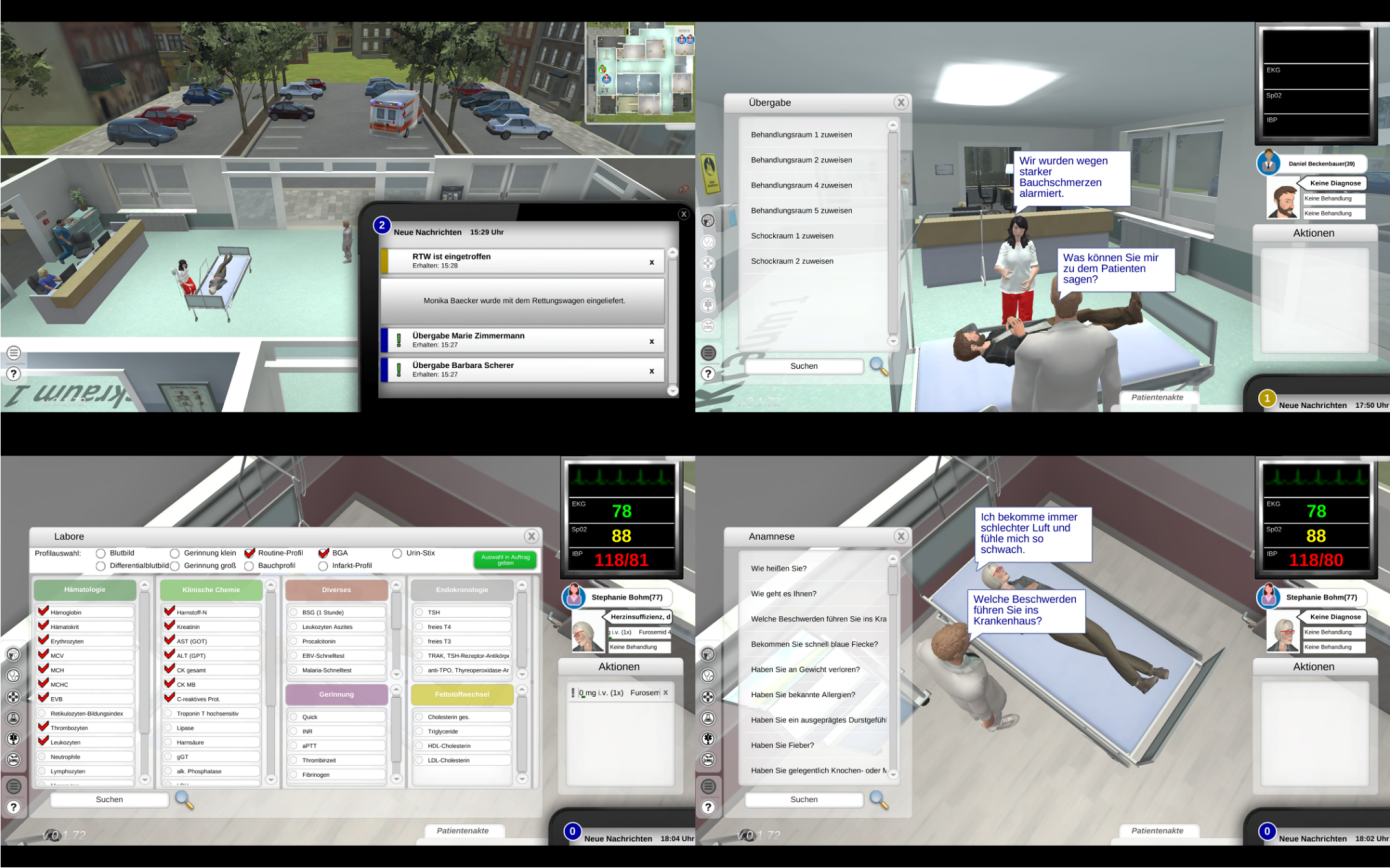
Screen shots of EMERGE. Top left, pager messages a new patient´s arrival. Top right, paramedic provides handoff information. Lower left, selection menu for laboratory tests. Lower right, selection menu for medical history. Graphics by PatientZero Games GmbH.

EMERGE is a complex computer-based virtual A&E department designed for undergraduate medical education (see Fig 1). Since 2012, it has been developed at Göttingen Medical School in collaboration with the University Medical Centre Hamburg-Eppendorf and PatientZero Games GmbH^*®*^. Players take on the role of the attending physician and are prompted to take medical histories, choose appropriate diagnostic tests, identify the most likely diagnoses and take adequate therapeutic measures while treating up to ten patients simultaneously. In contrast to traditional teaching formats, students playing EMERGE face the challenges of assessing the urgency of emergency situations, prioritising tasks and coordinating several activities at once. After having transferred a patient to a specific care unit, students receive a digital feedback on their diagnosis and treatment. Additionally, a senior physician´s recommendation on how to successfully solve the case is provided. In the summer term 2016, EMERGE was first implemented into the curriculum at Göttingen Medical School.

### Data collection and statistical analysis

One month before the beginning of the summer term 2016, students were informed about the study by e-mail. On the first day of the six-week repetition module, the study rationale was explained and students were invited to provide written consent to participate in the study and to have their data analysed.

In order to detect possible differences in performance between the PBL group and the EMERGE group in the final key feature examination, the achieved percentages of the maximum score were calculated and compared between the two groups (aggregate scores and subscores per case) using Mann-Whitney-U tests.

For the analysis of the final EMERGE session, all information contained in the log files was encoded and imported into IBM SPSS Statistics 24.0 (IBM Corp., Armonk, New York, USA). For each of the four cases that were presented during the final EMERGE session, the conditions for a successful patient treatment were determined based on national guidelines. Only data of cases that had been ‘closed’ (as indicated by students having transferred the patient to another care unit) were included in this analysis. First, an aggregate score was calculated from all student activities that were predefined as being appropriate based on guideline recommendations. Second, this score was broken down in subscores for the following categories: ‘correct history’, ‘correct physical examination’, ‘correct instrumental examination’, ‘correct laboratory orders’, ‘correct therapeutic interventions’, ‘correct diagnosis’ (dichotomous) and ‘correct patient transfer’ (dichotomous). Aggregate score and subscores were compared between the two study groups using Mann-Whitney-U or χ^2^ tests, as appropriate. Finally, student performance in each category was compared across cases.

All data analyses were performed using IBM SPSS Statistics 24.0 (IBM Corp., Armonk, New York, USA). The Kolmogorov-Smirnov test was used for assessing the normality of the given data. Results of descriptive analyses are presented as percentages (n) or means and interquartile ranges (IQR), as appropriate. Significance levels were set to 5%.

### Ethical approval

The local Institutional Review Board (Ethik-Kommission der Universitätsmedizin Göttingen; application number 18/3/16) waived ethical approval as the study protocol was not deemed to represent biomedical or epidemiological research. We made every effort to comply with data protection rules and all data were anonymized prior to analysis. Study participation was voluntary and all participants signed an informed consent form before entering the study.

## Results

### Participant characteristics

**Fig 2:**
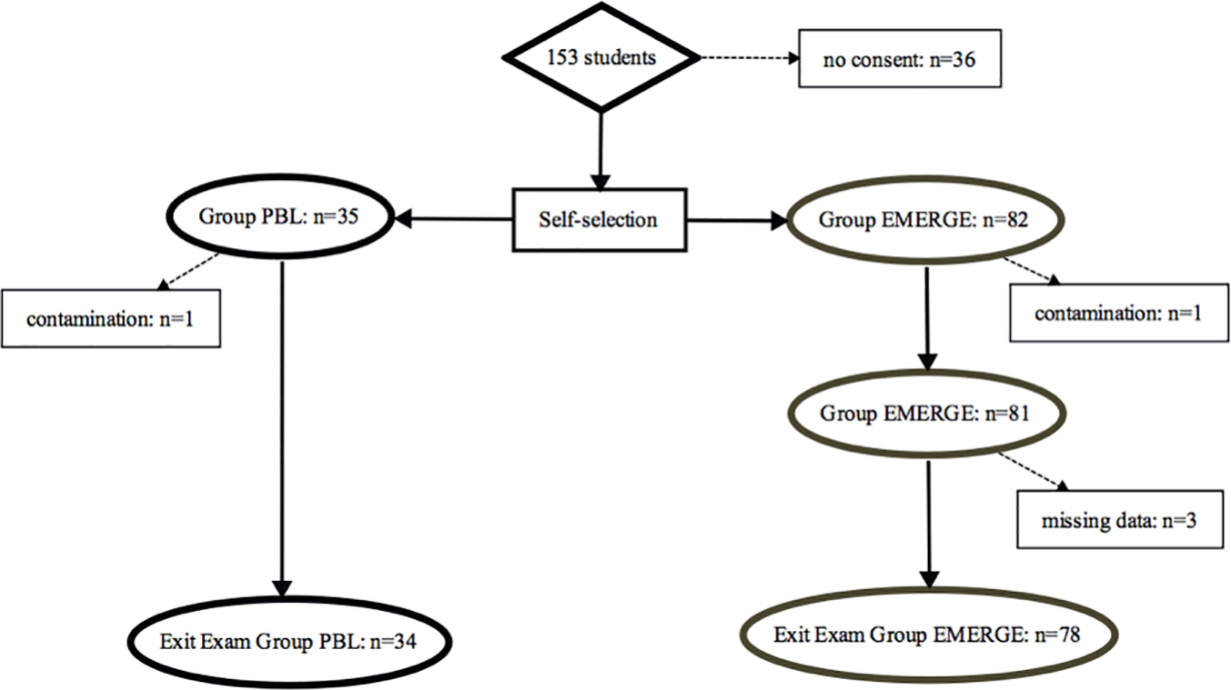
Flow of participants through the study. Contamination occurred when students attended another group´s teaching sessions and thereby were erroneously exposed to the wrong session at least once.

A total of 153 students were enrolled in the six-week repetition module, of which 117 provided written consent (see Fig 2). Thirty-five students self-selected into the PBL group and eighty-two students self-selected into the EMERGE group. Following the exclusion of students due to contamination or missing data, complete data were available for 34 students in the PBL group and for 78 students in the EMERGE group. Taken together, the effective response rate was 73.2% (112 out of 153 eligible students).

A total of 56.3% (n = 63) of the participants were female. There were no significant differences regarding sex distribution, age and previous performance levels at the ten training sessions between students in the PBL group and students in the EMERGE group (see Table 1).

**Table 1:** Participant characteristics. P values were derived from a Mann-Whitney-U-Test.

	PBL (n = 34)	EMERGE (n = 82)	P value
Female [%]	52.9 (n = 18)	57.7 (n = 45)	0.643
Age at the first training session, median (IQR) [years]	26.0 (5.0)	25.0 (3.0)	0.323
Examination scores during the previous semester, median (IQR) [%]	89.3 (4.6)	88.3 (6.6)	0.524

### Key feature examination

Table 2 presents student performance in the formative key feature examination as a function of exposure to EMERGE or PBL, respectively. Students in the EMERGE group achieved significantly higher aggregate scores than the PBL group (62.5 (IQR: 17.7)% vs. 54.2 (IQR: 21.9)%; p = 0.015). A case-by-case analysis revealed that students in the EMERGE group scored significantly higher than students in the PBL group regarding the two cases ‘heart failure’ (75.0 (IQR: 25.0)% vs. 50.0 (IQR: 25.0); p<0.001) and ‘hyponatremia’ (50.0 (IQR: 25.0)% vs. 50.0 (IQR: 31.3); p = 0.003). There was no significant difference in performance levels between the EMERGE group and the PBL group in the other four cases. This includes the cases ‘sarcoidosis’ and ‘Hodgkin lymphoma’ which had been discussed in both instructional formats during the training phase. Thus, a significant performance difference was found for two out of the four cases that were only presented in EMERGE sessions but not in PBL sessions.

**Table 2:** Student performance in the final exam part key feature.

	PBL (n = 34)	EMERGE (n = 78)
Percentage of maximum score (IQR)		
Total score	54.2 (21.9)	62.5 (17.7) *
Fever in aplasia	50.0 (25.0)	75.0 (25.0)
Heart failure	50.0 (31.3)	75.0 (25.0) ***
Hodgkin lymphoma	75.0 (25.0)	75.0 (25.0)
Hyperthyroidosis	50.0 (50.0)	50.0 (31.3)
Hyponatremia	50.0 (31.3)	50.0 (25.0) **
Sarcoidosis	50.0 (25.0)	50.0 (25.0)

*p < 0.05

**p<0.01

***p<0.001 in a Mann-Whitney-U-Test comparing the results of the PBL group and the EMERGE group.

### Final EMERGE session

Students in the EMERGE group achieved significantly higher aggregate scores than students in the PBL group in all four cases (NSTEMI: 68.4 (IQR: 15.8)% vs. 47.4 (IQR: 15.8)%; p<0.001; pancreatitis: 66.7 (IQR: 13.3)% vs. 46.7 (IQR: 13.3)%; p<0.001; gastrointestinal haemorrhage: 58.3 (IQR: 16.7)% vs. 41.7 (IQR: 16.7)%; p<0.001; asthma exacerbation: 66.7 (IQR: 16.7)% vs. 58.3 (IQR: 16.7)%; p = 0.004). Across cases, student scores in the EMERGE group compared to the PBL group were significantly more favourable in four of seven categories (final diagnosis, therapeutic interventions, physical examination, instrumental examination; see Table 3). For instance, students in the EMERGE group provided the correct diagnosis significantly more often than students in the PBL group in three of four cases.

**Table 3:** Student performance in the final EMERGE session.

	PBL	EMERGE
Total score: percentage of maximum score (IQR)		
NSTEMI	47.4 (15.8) (n = 27)	68.4 (15.8) (n = 74) ***
Pancreatitis	46.7 (13.3) (n = 26)	66.7 (13.3)(n = 73) ***
Gastrointestinal haemorrhage	41.7 (16.7) (n = 18)	58.3 (16.7) (n = 69) ***
Asthma exacerbation	58.3 (16.7) (n = 19)	66.7 (16.7) (n = 73) **
Correct diagnosis: dichotomous		
NSTEMI	70.4 (n = 19)	97.3 (n = 72) ***
Pancreatitis	46.2 (n = 12)	72.6 (n = 53) *
Gastrointestinal haemorrhage	33.3 (n = 6)	58.6 (n = 41) ***
Asthma exacerbation	84.2 (n = 16)	97.3 (n = 71) *
Correct therapeutic interventions: percentage of maximum score (IQR)		
NSTEMI	33.3 (33.3) (n = 27)	33.3 (41.7) (n = 74) **
Pancreatitis	0.0 (33.3) (n = 26)	33.3 (33.3) (n = 73) ***
Gastrointestinal haemorrhage	0.0 (0.0) (n = 18)	0.0 (0.0) (n = 69)
Asthma exacerbation	33.3 (33.3) (n = 19)	66.7 (33.3) (n = 73) ***
Correct history: percentage of maximum score (IQR)		
NSTEMI	50.0 (12.5) (n = 27)	50.0 (28.1) (n = 74)
Pancreatitis	50.0 (33.3) (n = 26)	50.0 (16.7) (n = 73) *
Gastrointestinal haemorrhage	50.0 (37.5) (n = 18)	50.0 (25.0) (n = 69)
Asthma exacerbation	50.0 (33.3) (n = 19)	50.0 (33.3) (n = 73)
Correct physical examination: dichotomous		
NSTEMI	81.5 (n = 22)	97.3 (n = 72) **
Pancreatitis	96.2 (n = 25)	98.6 (n = 72)
Gastrointestinal haemorrhage	66.7 (n = 12)	94.3 (n = 66) **
Asthma exacerbation	94.7 (n = 18)	91.8 (n = 67)
Correct instrumental examination: percentage of maximum score (IQR)		
NSTEMI	33.3 (0.0) (n = 27)	66.7 (33.3) (n = 74) ***
Pancreatitis	100.0 (100.0) (n = 26)	100.0 (0.0) (n = 73) ***
Gastrointestinal haemorrhage	0.0 (0.0) (n = 18)	100.0 (100.0) (n = 69) ***
Asthma exacerbation	-	-
Correct laboratory orders: percentage of maximum score (IQR)		
NSTEMI	100.0 (0.0) (n = 27)	100.0 (0.0) (n = 74)
Pancreatitis	66.7 (33.3) (n = 26)	100.0 (33.3) (n = 72) ***
Gastrointestinal haemorrhage	75.0 (50.0) (n = 18)	100.0 (50.0) (n = 69)
Asthma exacerbation	100.0 (100.0) (n = 19)	100.0 (100.0) (n = 73)
Correct patient transfer: dichotomous		
NSTEMI	77.8 (n = 21)	82.4 (n = 61)
Pancreatitis	53.8 (n = 14)	58.9 (n = 43)
Gastrointestinal haemorrhage	66.7 (n = 12)	90.0 (n = 63) *
Asthma exacerbation	15.8 (n = 3)	5.5 (n = 4)

*p < 0.05

**p<0.01

***p<0.001 in a Mann-Whitney-U-Test comparing the results of the PBL group and the EMERGE group.

Sample sizes vary because only students ‘closing’ a specific case were included in the analysis for the respective case.

## Discussion

To the best of our knowledge, this is the first prospective study comparing student learning outcome on CR as a function of exposure to a virtual A&E department (EMERGE) versus PBL.

The two main results of this study are the following: First, there was no performance difference in the key feature examination regarding those cases both groups had been exposed to during the study. Performance in the EMERGE group was significantly better in cases that had only been shown to students in this group. Second, as expected from the fact that students in the EMERGE group were more familiar with the game than students in the PBL group, performance in the final EMERGE session was significantly better in the EMERGE group.

According to the current literature, examinations using key feature questions are well-suited for the assessment of CR [23]. In the key feature examination, the EMERGE group scored significantly higher than the PBL group regarding the total score and two specific cases. Thus, the overall difference was driven by the difference in these two cases. Students in the EMERGE group but not in the PBL group had been exposed to these two cases, indicating that exposure to the content was associated with better learning outcome. This is important as learning time was similar in both groups. In fact, students in the PBL group might have spent more time on self-study between the sessions than students in the EMERGE group, introducing a certain bias in learning time favouring the PBL group. The finding of non-inferiority of EMERGE even for the two cases that were discussed in great depth in the PBL group is encouraging. Students in the EMERGE group worked through more than 40 different cases during the training phase whereas the PBL group only acquired in-depth knowledge about the five different cases they had spent ten sessions on. The fact that student performance on two of the four cases that were only presented in EMERGE was similar in the two student groups suggests that students had learnt how to approach the diagnosis and treatment of these diseases independent of the teaching format used in this study.

The two instructional formats differ with regard to the intended learning objectives: PBL fosters ‘deep learning’ [24] and the generation of detailed knowledge on specific diseases. Due to this approach, the total number of cases students can be exposed to during a ten-week training phase is very limited. Thus, the main difference between EMERGE and PBL is the number of cases that can be addressed in the same amount of time. It is one of the main findings of this study that EMERGE facilitates exposure to far more content in the same time without hampering learning outcome as measured in a key feature examination. We cannot comment on potential deficiencies regarding deep learning in the EMERGE group as we were not aware of any assessment method solely addressing the results of deep learning.

Another major difference between the two formats is that in PBL students do not need to treat various patients simultaneously. This means that key components of clinical decision making such as triaging, assessing the severity of illnesses and setting priorities can hardly be trained. EMERGE, in contrast, both demands and fosters these specific learning objectives. Our findings in the final EMERGE session show that students in the EMERGE group did not only demonstrate good clinical decision-making skills, but also good procedural skills. They achieved excellent results and successfully transferred their competencies regarding CR to unknown cases.

It is noted that the overall performance levels as indicated in the key feature examination and the final EMERGE session were moderate in both groups, with percentages being below 60% for the majority of items. This may be due to the fact that both assessments were formative in nature.

### Comparison with previous research

The findings of this study are in line with previous results regarding the effectiveness of Serious Games in medical education which, overall, show that Serious Games enhance learning compared with traditional methods [25–27]. However, careful consideration must be given to the fact that the existing studies vary greatly in terms of materials, measures, game design and game purpose which makes results difficult to compare and generalize [12]. Some studies indicate that Serious Games are at least as effective as traditional teaching formats regarding learning outcomes. Dankbaar et al. [18], for example, used a Serious Game for the teaching of knowledge and skills concerning patient safety. The study revealed that patient safety knowledge had equally improved both after using the game and studying with traditional text-based lectures in an e-module. Similar results were found, among others, for the acquisition of knowledge and skills on insulin therapy [28] as well as the development of team leadership and crisis management skills [29]. Other studies demonstrate that Serious Games may even be superior to traditional teaching formats concerning the improvement of complex cognitive functions. Better outcomes were shown for knowledge and skill acquisition on basic life support with an automated external defibrillator course [30] as well as on paediatric respiratory content [31], major incident triage [32] and cardiac examination competency [33]. However, it should be noted that a number of these studies focus on virtual patients [30,31,33] and that their results are not fully transferable to the current study. Therefore, comparisons should be made and interpreted with caution.

Additionally, these studies suffer from several limitations such as small sample sizes [28,29] or the use of the Serious Game as an additional, voluntary, extra-curricular activity [28,29,34] which may have confounded their results. Most importantly, the majority of published studies used multiple-choice questions to assess learning outcomes and, in particular, CR [35]. This type of assessment is more suitable for factual knowledge [36], and it is hard to write MC questions addressing higher-order cognitive functions such as the complex processes underlying CR. In order to assess problem-solving and clinical decision-making, assessment methods demanding these abilities such as key feature questions [23], which were also used in the current study, appear to be more appropriate. Our results indicate that EMERGE itself might be used as a (summative) assessment tool if students have sufficient opportunity to acquaint themselves with the functionality of the game before taking the exam.

### Strengths and limitations of the study

To our knowledge, this was the first study to evaluate the effectiveness of a Serious Game that was embedded in an official curriculum for undergraduate medical education. All cases referred to relevant problems of internal or emergency medicine and the response rate was favourable. However, the monocentric nature of this study and the selected group of fifth-year medical students limits the extent to which the findings can be generalized to other student groups and to content areas other than internal and emergency medicine. Additionally, the groups in this study were not randomized as students self-selected to take part in either EMERGE or PBL. A randomized design with a control group would have been desirable but it was not ethically justifiable to assign students to a novel, digital teaching format whose effectiveness was largely unknown. Moreover, assigning students to a novel teaching format against their will may have led to dissatisfaction resulting in an impaired motivation to participate in the current study and in confounding the results. Given that students self-selecting into the EMERGE group might have expressed their preference for digital resources, it remains to be seen if our results will replicate in a randomised setting where these preferences will not be accounted for. Given that the non-inferiority of EMERGE compared to PBL has now been established, future studies should be randomised in order to decrease validity threats. It might also be advisable to include a pre-test in order to separate previous student performance levels from learning outcome that occurred during (and, thus, is attributable to) the respective intervention. In addition, this would allow to evaluate the extent to which EMERGE and PBL enhance CR.

The current study showed that learning with EMERGE is at least as effective as PBL. However, according to Cook et al. [37] studies comparing computer-based learning to noncomputer instruction are usually difficult to interpret. The authors suggest that comparisons conducted within the same level of instructional design are more likely to produce meaningful and generalizable results. Thus, for future research, it would also be interesting to compare the effectiveness of EMERGE to other digital formats.

As stated above, learning time was a potential confounder in this study. We did not collect any data on the time the students spent on self-directed learning outside the teaching sessions. Due to this, the impact of independent student learning on the results remains unclear. This applies to all students regardless of their participation in EMERGE or PBL. However, it can be assumed that students in the PBL group spent more time on self-directed learning outside the teaching sessions as they were specifically asked to acquire detailed knowledge about the teaching content between the classroom sessions. Nevertheless, for future trials, we recommend gathering data on the average learning time spent on private study to evaluate its influence on the results.

Moreover, the teacher-student ratio differed between the two formats as it was 1:6 in the PBL group and 1:25 in the EMERGE group. In both formats, students were able to receive personal feedback during the sessions. In addition, EMERGE provided a short digital feedback on every case that was not aligned to student activity during the game. However, the data obtained in this study did not allow us to assess the extent to which students in the EMERGE group used the digital feedback. In future trials, appropriate data should be generated in order to facilitate a comparison of the type and amount of feedback provided in the two formats and its possible effect on student learning outcome regarding CR.

Regarding the final EMERGE session, students in the PBL group may have been less familiar with the software than students in the EMERGE group. Although the game´s navigation is quite intuitive and easy to learn, this disadvantage could have influenced the results. However, picking a diagnosis from a list on a computer screen does not require specific training, and yet we found significantly higher proportions of students providing a correct diagnosis for three of four cases in the EMERGE vs. the PBL groups. Nevertheless, it would be advisable for future studies to make use of an assessment format which is new and unfamiliar for both groups (e.g. a practical exam involving standardized patients) in order to avoid the possibility of giving an advantage to one of them.

The selection of key feature cases might have introduced a bias favouring the EMERGE group: Two cases were related to content that had been discussed in both formats during the training phase whereas four other cases referred to content that only students in the EMERGE group had been exposed to. Accordingly, students in the EMERGE but not the PBL group were encouraged to explore and study the latter cases during the training phase. Thus, the significantly better performance of students in the EMERGE group in the key feature cases ‘heart failure’ and ‘hyponatraemia’ may have been driven by their exposure to corresponding content during the training phase. More importantly, however, there was no significant difference regarding those two cases which had previously been discussed in both the EMERGE and the PBL group. As stated above, this is encouraging because the amount of exposure to different clinical cases in the EMERGE group (40 cases) was eight times higher than in the PBL group (5 cases). Thus, students in the EMERGE group spent less learning time on the content covered by those two key feature cases than students in the PBL group, but this did not have a negative impact on key feature exam results.

Finally, we used a formative assessment for the evaluation of learning outcomes. On the one hand, this type of assessment guaranteed that there was no incentive to be well-prepared for the examination in order to achieve a good mark which could have masked any true effect [38]; on the other hand, students both in the EMERGE and in the PBL group may have been less motivated to make an effort and to achieve high scores. Additionally, the formative assessment does not provide any insight into the intuitive and analytical processes involved in CR. While novices usually apply analytical processes, increasing experience in problem-solving and CR enables them to make use of the intuitive approach, as and where appropriate. Future research should address the impact of EMERGE and PBL on analytical and intuitive processes for example by using the think aloud method.

## Conclusion

Using a Serious Game is at least as effective as interactive PBL regarding CR training. One of the advantages of EMERGE is that students can be exposed to a high number of cases in short time without hampering learning outcome in specific cases. Further research is needed to investigate the mechanisms by which Serious Games may enhance clinical reasoning and how they can be used most efficiently in undergraduate and postgraduate medical education.

## Supporting information

S1 Dataset Full anonymized dataset. (SAV)
